# Mesenchymal stem cells in craniofacial reconstruction: a comprehensive review

**DOI:** 10.3389/fmolb.2024.1362338

**Published:** 2024-04-16

**Authors:** Zizhuo Zheng, Hanghang Liu, Shibo Liu, En Luo, Xian Liu

**Affiliations:** State Key Laboratory of Oral Diseases and National Center for Stomatology and National Clinical Research Center for Oral Diseases, West China Hospital of Stomatology, Sichuan University, Chengdu, Sichuan, China

**Keywords:** SuMSCs, iPSCs, ADSCs, PDLSCs, GMSCs, tissue engineering, craniomaxillofacial defecting reconstruction

## Abstract

Craniofacial reconstruction faces many challenges, including high complexity, strong specificity, severe injury, irregular and complex wounds, and high risk of bleeding. Traditionally, the “gold standard” for treating craniofacial bone defects has been tissue transplantation, which involves the transplantation of bone, cartilage, skin, and other tissues from other parts of the body. However, the shape of craniofacial bone and cartilage structures varies greatly and is distinctly different from ordinary long bones. Craniofacial bones originate from the neural crest, while long bones originate from the mesoderm. These factors contribute to the poor effectiveness of tissue transplantation in repairing craniofacial defects. Autologous mesenchymal stem cell transplantation exhibits excellent pluripotency, low immunogenicity, and minimally invasive properties, and is considered a potential alternative to tissue transplantation for treating craniofacial defects. Researchers have found that both craniofacial-specific mesenchymal stem cells and mesenchymal stem cells from other parts of the body have significant effects on the restoration and reconstruction of craniofacial bones, cartilage, wounds, and adipose tissue. In addition, the continuous development and application of tissue engineering technology provide new ideas for craniofacial repair. With the continuous exploration of mesenchymal stem cells by researchers and the continuous development of tissue engineering technology, the use of autologous mesenchymal stem cell transplantation for craniofacial reconstruction has gradually been accepted and promoted. This article will review the applications of various types of mesenchymal stem cells and related tissue engineering in craniofacial repair and reconstruction.

## 1 Introduction

The skull, composed of the frontal, parietal, interparietal, and occipital bones, provides critical protection for the brain and important sensory organs. The jaws are made up of the maxilla and mandible. Most of the craniofacial skeleton develops from the neural crest and forms through intramembranous ossification (Mundlos et al., 1997). However, the mandible differs in this regard; Parada and Chai found that the mandibular body forms through intramembranous ossification, while the condylar process forms through endochondral ossification (Park et al., 2016).

Craniofacial defects are complex lesions with a wide range of etiologies, including accidents, congenital conditions, pathology, or surgery. When the normal regenerative capacity of bone is exceeded or insufficient, bone grafting may be necessary. Surgeons have a variety of options for bone grafting, including the “gold standard” autologous bone graft. However, this method is not without drawbacks, such as morbidity, pain, infection associated with harvesting bone from donor sites, or poor bone quantity and quality in certain patient populations (Glowacki et al., 1981; Kawecki et al., 2018). Additionally, due to the specific and diverse morphology of craniofacial bones, autologous bone from donor sites may not perfectly fit bone defects. MSCs are cells that can be isolated from bone marrow, adipose tissue, dental pulp, umbilical cord and placenta, peripheral blood, and induced pluripotent stem cells. They have unique abilities of self-renewal and multilineage differentiation (Cai and Guo, 2023). MSCs are abundant and can be extracted autologously, cultured *ex vivo*, and then transplanted back into the body. Therefore, the use of mesenchymal stem cells (MSCs) in craniofacial reconstruction is currently a focus of research.

Tissue engineering is a promising approach to complement existing craniofacial bone regeneration treatment strategies. The classic core tissue engineering paradigm involves the integrated use of cells, biomaterial scaffolds, bioactive factors, and/or bioreactors. The main idea is to start with a biopsy of patient tissue, expand these primary cells in the laboratory, and then seed them into precisely tailored biomaterial scaffolds to create 3D tissues with the help of various biochemical (e.g., growth factor stimulation) and biophysical factors during bioreactor culture (Radisic and Alsberg, 2017). Tissue engineering provides a suitable growth space and differentiation environment for the transplantation of mesenchymal stem cells. For severe craniofacial defects, tissue engineering allows mesenchymal stem cells to better exert their differentiation capabilities.

## 2 Bone marrow mesenchymal stem cells

Bone Mesenchymal Stem Cells (BMSCs) are the most classically used MSCs for bone repair and regeneration, possessing exceptional multipotent differentiation capabilities (into cartilage, bone, and marrow adipocytes) and hematopoietic supportive abilities. The exploration of BMSCs for the repair of bone and cartilage defects has spanned decades, with numerous related reviews available. In recent years, advancements in tissue engineering technology have renewed researchers’ interest in BMSCs. Therefore, this article will focus on the role of classical BMSCs in craniofacial defects and recent research progress on the application of BMSCs in craniofacial reconstruction (Table 1).

**TABLE 1:** The authors, materials, experimental subjects, experimental models, types of experiments, sources of mesenchymal stem cells, effects, and follow-up time of the literature on tissue engineering related to BMSCs.

Author	Scaffold	Research subjects and quantity	Diseases	Study design	MSCs source	Outcomes	Follow-up
Rajan et al. (2014)	β-TCP	human 1	Jaw defects caused by trauma	Case report	Autologous	80% of bone defects were regenerated, and the function and appearance of the patients were restored by implants	16 months
Kaigler et al. (2015)	β-TCP	humans 30	Maxillary sinus bone defect	Phase 1/2 RCT	Autologous	Accelerated bone regeneration; increased bone density	16 months
Quarto et al. (2001)	Macroporous HA scaffolds	humans 3	Large bone defect	Case report	Autologous	Callous formation, good integration with host bone, no complications	15–27 months
Behnia et al. (2012)	HA/TCP polygonal 3 mm cubes mixed with PDGF and SSCs	humans 3	Alveolar cleft defect	Case series	Autologous	51.3% fill of the bone defect	3 months
Kaigler et al. (2013)	Absorbable gelatin sponge	humans 24	Alveolar bone defect	Phase 1/2 RCT	Autologous	Accelerated alveolar bone formation; reduced need for secondary bone grafting	1 year
Wakitani et al. (2011)	Type I bovine collagen sheet	humans 41	Cartilage defect	Case series	Autologous	Cartilage regeneration without infection	75 months
Baba et al. (2016)	3D Woven-Fabric Composite Scaffold Gel	humans 10	Chronic periodontitis	Phase 1/2 RCT	Autologous	Decreased clinical attachment loss; reduced periodontal pocket depth	3 years
Aghali and Arman (2020)	visible light-cured thiol-acrylate hydrogels	mice	Skull defect	Animal experiments	Allogenic	Improved survival rate after transplantation, Enhanced differentiation capacity	21 days
Wang et al. (2018)	β-TCP	dogs 4	Alveolar ridge defects in dogs	Animal experiments	Autologous	Increased amount of newly formed bone	12 weeks
Roskies et al. (2016)	PEEK	rat	Craniomaxillofacial defects	Animal experiments	Autologous	Enhanced expression of chondrogenic differentiation	28 days
Li et al. (2020b)	β-TCP	Dogs	Alveolar bone defect	Animal experiments	Autologous	Improved bone density and enhanced osseointegration in the sinuses	12 months
Li et al. (2017)	CB-B-P	Mice 33	Craniomaxillofacial bone defect	Animal experiments	Autologous	Rapid neovascularization	12 weeks
Sun et al. (2017)	SCS	Mice 60	Fibula defect	Animal experiments	Autologous	Increased neovascularization	3 months

TCP, tricalcium phosphate; β-TCP, Beta-tricalcium phosphate; HA, hydroxyapatite; PEEK, polyetheretherketone; CB-B-P, chondrocyte brick-enriched platelet-rich plasma gel and BMSCs.

### 2.1 BMSCs enhance craniofacial osteogenesis and healing

Bone Mesenchymal Stem Cells (BMSCs), derived from the bone marrow as genome-wide stem cells, possess the capability to differentiate into mesoderm cells. They exhibit inherent advantages in osteogenic differentiation and possess excellent immunomodulatory potential. Studies have shown that the use of cells from the same embryonic origin in the process of reconstructing damaged bones significantly improves bone regeneration and formation (Akintoye et al., 2008; Leucht et al., 2008). As they can be extracted from the same site as the bone defect, such as the skull, mandible, craniofacial bones, and alveolar bones, BMSCs can provide targeted assistance in craniofacial bone reconstruction based on the location and severity of the defect (Behnia et al., 2012). Compared with traditional mechanically guided bone regeneration, injecting BMSCs into bone defects to promote bone regeneration causes less secondary damage to the bone defects and has higher applicability. Many researchers have found that after injecting BMSCs from the corresponding site into the jaw bone defects, the jaw bone regeneration speed increases and has higher bone density (Kaigler et al., 2013). In addition, some scholars have further transplanted BMSCs into bone defects or inflammatory sites using designed three-dimensional scaffold structures, thereby further improving the level of bone recovery, which is of great significance for the recovery of periodontitis, dental implants, and alveolar bone (Quarto et al., 2001; Kaigler et al., 2015; Baba et al., 2016).

There are differences in the roles of BMSCs from different sources in craniofacial bone reconstruction. Studies have shown that compared to other BMSCs, skull-derived BMSCs have higher osteogenic differentiation capabilities (Li C. et al., 2020; Hong et al., 2020). However, when applied to craniofacial fractures, BMSCs derived from alveolar bone may form cartilaginous callus at the fracture site, affecting normal bone healing (Matsubara et al., 2005). This indicates that BMSCs from different sources have different differentiation trends. If this can be utilized effectively, BMSCs will have broader application prospects. In fact, Horwitz EM et al. have attempted to use allogeneic BMSCs infusion for the treatment of hereditary skeletal diseases, and found significant effects in the treatment of congenital skull hypoplasia, craniosynostosis, and craniometaphyseal dysplasia (Horwitz et al., 2002).

Currently, transplanting BMSCs through tissue engineering to bone defect sites to assist in bone formation is considered a minimally invasive and efficient treatment method. Researchers use various spatially patterned three-dimensional materials to provide a more suitable environment for the growth and differentiation of BMSCs at bone defect sites (Rajan et al., 2014; Roskies et al., 2016; Wang et al., 2018), promoting the proliferation and migration of BMSCs, or promoting the expression of osteogenic-related genes in BMSCs, such as AMPK-Mfn1 (Fan et al., 2019), circ-CTTN, ROS/JNK, *etc.* (Li et al., 2022; Zhang M. et al., 2023). Tissue engineering technology has broadened the application fields and scopes of BMSCs, improved their promoting effect on jawbone reconstruction and repair, and provided new strategies for clinical treatment.

### 2.2 BMSCs enhance the attachment of implants to bone

Osteointegration refers to the direct contact between the implant and bone tissue, presenting a non-fibrous connective tissue interface layer observed under an optical microscope. Factors such as inflammation and insufficient bone density can lead to poor osteointegration, resulting in the failure of dental implants. Meanwhile, the effectiveness of bone density determines the outcome and lifespan of dental implants. Researchers have found that BMSCs play a promoting role in alveolar bone formation and osteointegration in the jawbone.

BMSCs play a crucial role in the process of alveolar bone formation and osseointegration. Injecting BMSCs into the bone graft area can promote an increase in bone density in that region, while also facilitating good osseointegration between the implant and the alveolar bone. This, in turn, enhances the tight bonding between the implant and the alveolar bone, reducing the risk of alveolar bone cracking or resorption after implant placement (Kaigler et al., 2013). Based on this, researchers continue to explore other methods to enhance the effects of BMSCs, such as co-injection with metformin or the use of various inducing factors to improve the osteogenic promotion effects of BMSCs in the injection area, leading to increased new bone formation within the alveolar bone. This may be related to the ability of these drugs to promote the expression of AMPK signaling proteins such as Runx2 in BMSCs (Lin et al., 2020). Zou utilized hypoxia-inducible factor-1α (HIF-1α) to transduce canine BMSCs and found that BMSCs expressing HIF-1α significantly promoted the formation of new bone in the peri-implant bone defect area and significantly enhanced osteointegration (Zou et al., 2012). Customized modulation of BMSCs to achieve bone formation promotion for different jaw and facial implant bone defects may be a major research direction in the future.

For more severe jawbone defects, tissue engineering technology can assist BMSCs in achieving more challenging bone repairs and osteointegration with implants (Aghali and Arman, 2020; Li Q. et al., 2020). As mentioned earlier, β-TCP still plays an active role in promoting osteointegration of BMSCs. Ma applied dentin matrix protein-1 (DMP1)-transduced BMSCs to tissue engineering using β-TCP as the material and found improved bone density and significantly enhanced osteointegration in the sinus area (Ma et al., 2020). Gjerde implanted BMSCs together with biphasic calcium phosphate granules into the alveolar bone of patients with severe alveolar ridge resorption who did not meet the implantation conditions. After 12 months, a large amount of new alveolar bone was generated, with sufficient bone volume and density to meet the conditions for dental implantation (Gjerde et al., 2018). Li et al. implanted graphene oxide (GO) together with implants and BMSCs into the alveolar bone and found a significant improvement in the osteogenic differentiation ability of BMSCs. It also promoted the polarization of BMSC-differentiated macrophages towards the M2 phenotype, reducing the expression of inflammation (Gjerde et al., 2018).

### 2.3 BMSCs promote angiogenesis independently or under the pre-vascularized scaffold environment

Compared to the maxilla, the mandible has a relatively limited blood supply. In cases of mandibular fractures or bone defects, restoring vasculature and blood flow is crucial to prevent complications such as osteonecrosis. Furthermore, even in areas with abundant blood supply like the maxilla and skull, the quantity and rate of neovascularization play a pivotal role in craniofacial reconstruction, especially in cases of comminuted craniofacial fractures. Bone marrow stromal cells (BMSCs) from different sources exhibit significant differences. Compared to BMSCs derived from the iliac crest, maxillary BMSCs demonstrate stronger angiogenic potential both *in vitro* and *in vivo*. This is because the ilium and jaw bone originate from different germ layers, and BMSCs located in the maxilla have higher expression levels of bFGF protein, leading to superior angiogenic potential (Du et al., 2016). Additionally, the expression of SDF-1α, TGF-β1, VEGFα, and BDGF-BB closely affects the angiogenic capacity of BMSCs (Sun et al., 2017). These factors can be promoted either through special materials, such as collagen scaffolds capable of sustained release of substances like silicic acid, or through co-culture with other cells - particularly adipose-derived stem cells (ASCs) - which stimulate BMSCs to secrete more angiogenesis-related factors like VEGFα and CXL1, suggesting a novel strategy for promoting angiogenesis (Kim et al., 2014).

Insufficient blood supply remains a significant challenge in tissue engineering. Unlike direct MSC transplantation, scaffolds used in tissue engineering create a distance between MSCs and the nearest capillaries, making it difficult for cells to survive beyond 200 μm (Zhang et al., 2012). Consequently, researchers aim to harness the angiogenic potential of BMSCs to rapidly form vasculature and support tissue regeneration. Some researchers have considered abandoning the scaffold structure and directly utilizing BMSCs and their differentiated endothelial cells to construct differentiated osteoblast sheets and prevascularized cell sheets as substitutes for scaffolds (Xu et al., 2019). This approach aims to create a scaffold-free structure that enhances the survival rate of transplanted BMSCs while ensuring their tissue regeneration function. Other researchers have focused on the temporal aspects of scaffolds, attempting to design scaffolds that can be absorbed and degraded in the body at appropriate times. This ensures that BMSCs can proliferate, migrate, and generate blood vessels smoothly after being implanted. Platelet-rich plasma gels perfectly meet this requirement as they not only eliminate gaps with capillaries after reaching the intended site but also stimulate the angiogenic capacity of transplanted BMSCs. Additionally, they help create a favorable microenvironment for bone repair (Li et al., 2017). Lu, on the other hand, chose to use BMSC-derived exosomal miR-29α instead of BMSCs for injection into mice, thus eliminating the need for a scaffold, and found that miR-29α could be taken up by human umbilical vein endothelial cells (HUVECs), promoting their proliferation, migration, and angiogenic capacity (Lu et al., 2020). Additionally, allowing BMSC-secreted exosomes carrying HIF-1α to enter the body independently can also promote angiogenesis (Ying et al., 2020).

### 2.4 BMSCs restore craniofacial-specific cartilage

In craniofacial reconstruction surgeries, the unique shape and structure often result in significant damage to the cartilage tissue during transplantation, making it challenging to meet the required amount of cartilage for the procedure (Calvert et al., 2014; Hsiao et al., 2014). Additionally, due to the avascular nature of cartilage, the risk of ischemia-induced necrosis following transplantation is higher compared to other tissues (Yoon et al., 2020). As a result, autologous transplantation of BMSCs to induce cartilage formation has emerged as an alternative to traditional cartilage transplantation methods. Transforming Growth Factor-β (TGF-β) is a key factor in chondrogenic differentiation of BMSCs. Exposure of BMSCs to TGF-β for 24 h *in vitro* prior to implantation can trigger chondrogenesis *in vivo* (Futrega et al., 2021), and high expression of TGF-β has been detected in BMSCs undergoing chondrogenic differentiation *in vivo* (Freyria and Mallein-Gerin, 2012). In addition, inhibiting Phosphatidylinositol-specific Phospholipase Cγ1 (PLCγ1) can enhance the chondrogenic capacity of BMSCs (Matta and Mobasheri, 2014; Chen et al., 2020). Furthermore, regulating the expression of related adipokines using drugs or other substances can also promote the chondrogenic differentiation of BMSCs. Wang discovered that the adipokine Vaspin can activate Akt expression, thereby activating the PI3K/Akt signaling pathway and promoting chondrogenic differentiation of BMSCs. This process also reduces the degradation of the extracellular matrix (ECM) and improves the survival rate of the formed cartilage (Wang et al., 2022). Zheng et al. found that the circular RNA (circRNA) ATRNL1 can upregulate the expression of cartilage factors such as COL2, SOX9, and Aggrecan, promoting chondrogenic differentiation of BMSCs (Zheng JZ. et al., 2021).

Researchers have also attempted to utilize tissue engineering techniques to assist in the chondrogenic differentiation of BMSCs. Specific scaffold structures can reduce the differentiation of BMSCs towards angiogenesis and osteogenesis, promoting their differentiation towards chondrogenesis (Wakitani et al., 2011). For example, in the study by Li et al., BMSCs transplanted using the CB-B-P system gradually underwent chondrogenesis after implantation in the nasal region, facilitating the regression of vascularization and favoring sustained chondrogenesis. (Li et al., 2017). Chen et al. used a PLCγ1 inhibitor to construct U73122+BMSCs, which were transplanted into rats via scaffolds. They found that this approach was more effective in repairing cartilage defects compared to transplantation of BMSCs alone (Chen et al., 2020). Interestingly, BMSCs can also enhance the survival and chondrogenic capacity of chondrocytes. Yuan injected extracellular vesicles (EVs) secreted by BMSCs into the nasal cavities of mice and observed that EVs could suppress chondrocyte apoptosis through miRNAs, improving chondrocyte survival under ischemic conditions (Yuan et al., 2022).

## 3 Suture mesenchymal stem cells

**FIGURE 1:**
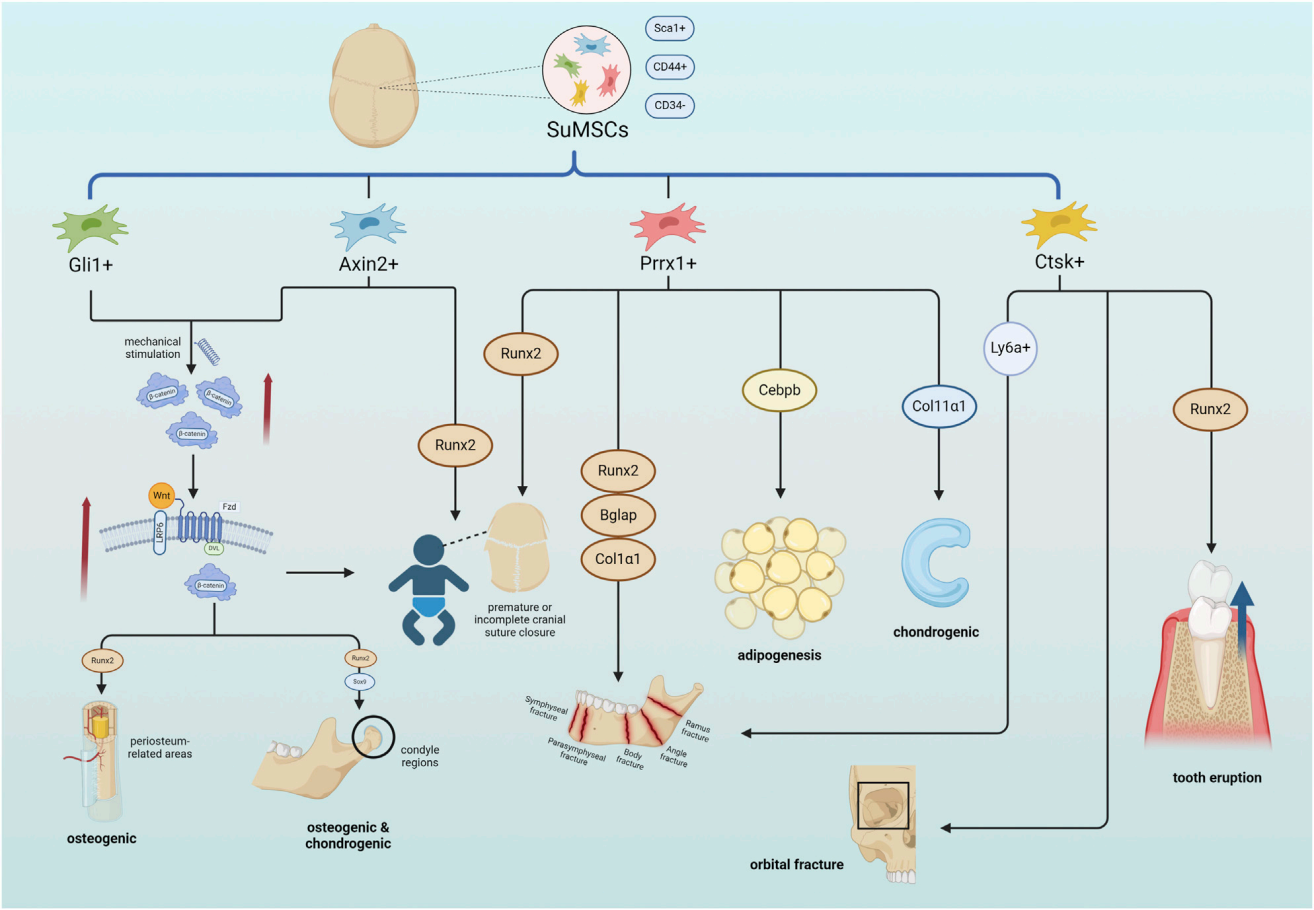
SuMSCs cells derived from cranial sutures can be divided into four types of SuMSCs according to different gene expressions: Gli1+, Axin2+, Prrx1+, and Ctsk+. Both Gli1+SuMSCs and Axin2+SuMSCs promote the expression of β-catenin in response to tensile mechanical stimulation, thereby activating the Wnt pathway and facilitating the normal closure of neonatal craniofacial sutures. Gli1+SuMSCs can also increase Runx2 expression in the periosteal region by activating the Wnt pathway to promote osteogenesis, while in the condylar part, it can simultaneously increase Runx2 and Sox9 expression to promote osteogenesis and chondrogenesis. Both Axin2+SuMSCs and Prrx1+SuMSCs can increase Runx2 expression to directly treat neonatal craniosynostosis or insufficiency. Prrx1+SuMSCs can also increase the expression of Runx2, Bglap, and Col1α1 to promote bone repair in mandibular defects, increase Cebpb to promote facial adipogenesis, and increase Col11α1 to promote condylar and nasal cartilage formation. Ctsk + SuMSCs can promote bone healing in orbital fractures, and can also regulate alveolar bone density by expressing Runx2 to ensure normal tooth eruption. Ctsk + SuMSCs expressing the Ly6a gene can specifically promote mandibular bone formation References (Created with BioRender.com).

There are closely connected craniofacial sutures formed by a small amount of connective tissue, which are not only the connection between various parts of the skull, but also an important ecological niche for osteogenesis. Suture mesenchymal stem cells (SuMSCs), a class of stem cells that only exist in the mesenchyme of the cranial suture. SuMSCs have different differentiation capacities and fates at different positioning of the suture, Lana-Elola et al. pointed out that SuMSCs closer to the midline of the suture mesenchyme have less tendency to differentiate (Lana-Elola et al., 2007). Up to now, four subsets of SuMSCs have been identified, namely, Gli1+, Axin2+, Prrx1+ and Ctsk+(Figure 1).

### 3.1 Gli1+ SuMSCs promote craniofacial bone and cartilage regeneration

The Hedgehog-Gli signaling pathway, which has been established as crucial for tissue development and homeostasis, involves Gli1 as a marker closely associated with craniofacial osteogenesis. Cells expressing Gli1 have been shown to promote cranial bone formation and craniofacial repair. Guo et al. employed cell flow cytometry to demonstrate that craniofacial Gli1+ cells express high levels of markers specific to mesenchymal stem cells (MSCs), such as Sca1 and CD44, and do not express CD34, suggesting that these cells are predominantly MSCs (Guo et al., 2018). K Amano et al. distinguished Gli1+ and Gli1-chondrocytes in the cranial base using Gli1-CreERT2; Tomatofl/+ and Gli1-CreERT2; Pth1rfl/fl; Tomatofl/+ mice and validated the crucial role of Gli1+ cells in early cranial development and adulthood through the Pth1r signaling pathway (Amano et al., 2023). Zhang et al. also studied the distribution of Gli1+ cells in the mandible using Gli1-CreERT2 mice and found that Gli1+ cells are present in specific regions of the periosteum and condyle, with stronger proliferative capacity in the condyle region (Zhang N. et al., 2023). In terms of differentiation potential, Gli1+ cells show significant differences depending on their location; they possess both osteogenic and chondrogenic potential in the condyle region, while only expressing osteogenic potential in periosteum-related areas. This suggests that the growth and remodeling capacity of the condyle is significantly stronger than that of the body of the mandible.

For patients with craniofacial bone dysplasia, minimally invasive craniotomy with expansion spring assistance has gradually replaced traditional craniotomy in recent years, providing higher safety and better tissue recovery (van Veelen and Mathijssen, 2012; van Veelen et al., 2018). The therapeutic principle of this surgery is that the edges of cranial sutures are stimulated by tensile force to show an osteogenic trend. The reason is that Gli + SuMSCs are highly sensitive to mechanical force. They rapidly proliferate and exhibit a significant osteogenic trend within 3 days after exposure to tension, greatly promoting bone formation at the suture site (Jing et al., 2022). Conditional knockout of β-catenin in Gli1+ SuMSCs, however, severely limits their activation and thereby limits bone reconstruction after tensile expansion. Shi et al. also demonstrated, using a fracture model in Gli1-CreERT2 mice, that Gli1+ cells contribute to both osteogenesis and chondrogenesis during fracture healing (Shi et al., 2017). The number of Gli1+ cells was significantly increased compared to non-fractured areas, suggesting that Gli1+ cells may expand rapidly after fracture to promote bone healing. Similarly, Huang utilized two transgenic mouse models that labeled Gli + cells to investigate the role of Gli + cells under mechanical force stimulation using a rapid maxillary expansion (RME) mouse model and a mechanical stretching loading cell model. They found that the calcium ion channel IP3R located on the endoplasmic reticulum was regulated by Gli + cells to promote osteogenesis. This study demonstrated that Gli1+ cells can sense mechanical forces and regulate calcium ion channels to modulate osteogenesis (Huang et al., 2021). These reveal the specificity of Gli1+ in the process of skull reconstruction, providing a direction for skull reconstruction.

### 3.2 Axin2+ SuMSCs ensure proper growth of cranial sutures and bone healing

Axin2+ cells possess the capabilities of long-term self-renewal, clonal expansion, and differentiation during skull development, indicating that Axin2 may serve as a suitable specific marker for skull stem cells. Tracking and observing these cells may facilitate the analysis of the stages and underlying causes of skull developmental defects (Maruyama et al., 2017). Simultaneously, Axin2 is expressed in the periosteum and endosteum within the osteogenic niche of the human skull, and its expression in the skull is significantly higher than that in long bones. This evidences a unique role for Axin2 in the formation and differentiation of the skull, as mutations in Axin2+ cells can lead to premature closure of skull sutures (Di Pietro et al., 2020).

In addition, since the sutures are formed by connecting fibrous joints known as sutures, premature fusion of these fibrous joints can lead to cranial dysplasia. Elanur Yilmaz believes (Yilmaz et al., 2018)that this may be related to incomplete intramembranous ossification mediated by the Wnt signaling pathway. Axin2 has been shown that Axin2 negatively regulates the Wnt pathway and coordinates the regulation of β-catenin during osteogenesis in the cranial periosteum (Yu et al., 2005), thereby ensuring normal cranial development. In a comprehensive study, Meghan E reported that the downregulation of Axin2 expression stimulates the expression of Runx2 at the cranial sutures, resulting in prolonged β-catenin signaling and consequent promotion of osteogenesis. Furthermore, it was observed that Hdac3 effectively represses the transcriptional activity of Axin2, leading to the upregulation of Runx2 expression (McGee-Lawrence et al., 2013). In mice, Wnt-responsive cells (WRCs) expressing the Axin2 gene are activated after bone injury and generate bone and cartilage, further demonstrating that WRCs expressing Axin2 can serve as effective targets for skull reconstruction (Ransom et al., 2016).

In summary, modulating the expression of Axin2 in suture mesenchymal stem cells can effectively control the timing of cranial suture closure and promote osteogenesis through multiple pathways, which is of great significance for cranial reconstruction in patients with premature or incomplete cranial suture closure. This suggests that in the treatment of patients with premature or incomplete cranial suture closure, we can use drugs or other methods to regulate the expression of Axin2 gene for adjunctive therapy, which may improve the treatment effect and efficiency. However, there is currently limited research on clinical drugs that regulate Axin2, which may be a direction for future research.

### 3.3 Prrx1+ SuMSCs repair the dermis and promote adipogenesis

The expression of Prrx1 is widespread in the coronal suture at birth and remains exclusive to this location postnatally, serving as a marker for SuMSCs (Takarada et al., 2016; Farmer et al., 2021). Distinct from other SuMSCs, Prrx1+ cells exhibit a more diverse and versatile expression profile in the craniofacial region. Liu et al. demonstrated using Prrx1-Cre and Prrx1-CreERT2 mice that Prrx1+ cells not only contribute to bone formation but also possess the ability to generate white adipose tissue and dermis (Liu et al., 2022). Upon reduction of Prrx1+ cells, osteoporosis, decreased fat accumulation, and thinning of the dermal layer were observed, which hindered bone healing and scar formation at fracture sites. Furthermore, Prrx1+ cells maintain stem cell differentiation properties upon transplantation *in vitro*, suggesting their potential for craniofacial reconstruction. Jiang’s study on continuously stretched jawbone tissue revealed that Prrx1+ cells were actively engaged in osteogenic differentiation and expressed neural crest markers, consistent with the developmental origin of cranial bones (Jiang et al., 2023). This suggests that Prrx1+ cells effectively promote jawbone formation and regeneration during jawbone distraction osteogenesis. Enhancing Prrx1 expression may serve as an effective adjunctive therapy for craniofacial reconstruction in the future.

Tooze identified a significant enrichment of rare Prrx1 gene variants or deletions in patients with craniosynostosis or premature cranial suture closure, often with severe symptoms (Tooze et al., 2023). A follow-up of these patients’ families revealed that Prrx1 gene variants are heritable, highlighting the importance of regular examinations and monitoring of cranial bone status in individuals with pathogenic Prrx1 mutations and their families. However, the specific phenotypic presentation of patients with Prrx1 gene mutations differs from that observed in monogenic disorders (Albers et al., 2013), indicating the need for further exploration of the various mutations within the Prrx1 gene.

### 3.4 Ctsk + SuMSCs aid in the regeneration of orbital bone and mandibular angle bone

Cathepsin K (Ctsk), a collagenase specifically expressed in osteoclasts, is closely associated with bone resorption. Consequently, mutations in the Ctsk gene often lead to craniofacial diseases, such as dense bone hyperplasia characterized by open fontanelles and sutures. Chen et al. reported that Ctsk−/− mice exhibit symptoms including cranial suture separation, loss of mandibular angle, osteosclerosis, and increased bone fragility (Chen et al., 2007). Utokpat described a rare case of osteoblast proliferation syndrome resulting from a mutation in the coding region of Ctsk, manifesting as osteosclerosis, bone fragility, and skull deformities (Utokpat et al., 2013). Additionally, Otaify reported eight cases of consanguineously married individuals with pseudocystosis, all exhibiting the aforementioned typical manifestations and harboring distinct Ctsk gene mutations (Otaify et al., 2018).

The skeletal structures of various craniofacial regions exhibit significant morphological differences. Unlike the predominantly osteoclastic phenotype observed in long bones (Zenger et al., 2010), Ctsk + cells possess specific functions in different craniofacial locations, encompassing both osteoclastic and osteogenic capabilities. For instance, heterogeneity exists among Ctsk + cells in the mandibular bone marrow, where the Ctsk + Ly6a + subpopulation possesses migratory and osteogenic differentiation potential, responding to mandibular bone defects and thus playing a pivotal role in mandibular healing (Ding et al., 2022). In the orbital bone of the eye socket, Ctsk + cells are only abundantly expressed during the process of orbital bone repair and reconstruction, accompanied by osteogenic differentiation (Liu et al., 2023).

Within the oral cavity, Ctsk−/− mice demonstrate a notable reduction in jaw bone trabeculae and abnormal alveolar bone height compared to normal mice (Okaji et al., 2003). Similarly to other SuMSCs, Ctsk + cells regulate Runx2 gene expression, thereby controlling osteoclast differentiation and bone remodeling. Xin et al. observed mutations in Runx2 following the inhibition of the Ctsk axis, leading to impeded osteoclast differentiation and bone remodeling, resulting in blocked tooth eruption pathways (Xin et al., 2020). The absence of the Ctsk gene disrupts the delicate balance between osteoclastic and osteogenic functions. However, this also suggests that appropriate modulation of Ctsk expression during craniofacial reconstruction may promote bone healing and enhance craniofacial reconstruction outcomes.

## 4 Adipose-derived stem cells

Adipose-derived stem cells (ADSC) are multipotent stem cells derived from adipose tissue, which are easily extracted with minimally invasive procedures and possess excellent growth dynamics and plasticity. They have been demonstrated to be efficiently inducible into various cell types for tissue regeneration, including osteogenesis, chondrogenesis, and wound healing, which aligns well with craniofacial repair and reconstruction. Therefore, ADSCs are currently considered the most promising mesenchymal stem cells for craniofacial reconstruction applications (Paduano et al., 2017).

Due to the ubiquitous presence of fat in the body, ADSCs extracted from different anatomical sites may exhibit variations. Lin et al. found that CD34^+^ ADSCs possess multipotency of mesenchymal stem cells (Lin et al., 2008; Corselli et al., 2012) and exhibit stronger proliferative capacity, while CD34^−^ ADSCs demonstrate enhanced plasticity (Suga et al., 2009; Bailey et al., 2010).. (Table 2)

**TABLE 2:** The authors, materials, experimental subjects, experimental models, types of experiments, sources of mesenchymal stem cells, effects, and follow-up time of the literature on tissue engineering related to iPSCs.

Author	Scaffold	Research subjects	Diseases	Study design	iPSCs source	Outcomes	Follow-up
Togliatto et al. (2016)	exosome/β-TCP combination	Mice	Skull defect	Animal experiments	hiPSCs	Accelerated new bone formation	8 weeks
Lin et al. (2008)	collagen sponge scaffolds	Rats	calvarial defects	Animal experiments	mouse-iPSCs	Enhanced osteogenic capacity	4 weeks
Sándor et al. (2014)	Bioceramic akermanite	Mice	calvarial defects	Animal experiments	hiPSCs	Stimulate osteogenic differentiation and enhance angiogenesis	8 weeks
Dong et al. (2021)	biodegradable PGA	Rats	Vascular remodeling defects	Animal experiments	hiPSCs	Aorta regained good patency	6 weeks
Li et al. (2018)	micro-cavitary hydrogel platform	Mice	-	*In vitro* culture	mouse-iPSCs	Induced spongy reticulated cartilage	5 weeks
Yagi et al. (2004)	mechanic polycaprolactone/gelatin scaffolds	Mice	Cartilage defect	Animal experiments	mouse-iPSCs	Enhanced chondrogenesis	6 weeks

β-TCP, Beta-tricalcium phosphate; PGA, polyglycolic acid; PLGA, poly-lactic-co-glycolic acid.

### 4.1 ADSCs assist in restoring fullness to the maxillofacial region

The craniofacial region plays a crucial role in the aesthetic appearance of patients. Therefore, when performing craniofacial reconstruction, attention should be paid to restoring facial fullness, which requires the restoration of lost muscle and fat, as well as special cartilage tissues such as nasal cartilage. Adipose-derived stem cells (ADSCs) possess strong adipogenic differentiation capacity, conferring unique advantages in facial fat reconstruction, thus helping patients achieve better degrees of facial restoration. Additionally, the pluripotency of ADSCs endows them with the potential to differentiate into chondrocytes. However, due to the prominent adipogenic differentiation tendency of ADSCs, special material structures are required to induce their chondrogenic ability. Mäenpää combined adipose mesenchymal stem cells with their bilayer biodegradable polylactic acid (PLA) disks for chondrogenic induction culture and found that substances such as proteoglycans, type I collagen, and type II collagen, which are present in TMJ cartilage formation, were all detected in the culture medium (Mäenpää et al., 2010). Similar to the previously mentioned BMSCs, exosomes produced by ADSCs can also exert effects comparable to those of ADSCs, promoting bone regeneration. (Togliatto et al., 2016; Li et al., 2018), while also reducing osteoclastogenesis, protecting articular cartilage, and exerting anti-inflammatory effects by inhibiting the expression of RANKL.

In addition to the conventional ADSCs that can be extracted from adipose tissue, there are also fibroblast-like cells that are free of fat, known as dedifferentiated adipose (DFAT) cells (Yagi et al., 2004). Under appropriate conditions, DFAT cells can also possess proliferative capacity and differentiate into various cell types (Matsumoto et al., 2008). Tateno found that DFAT cells demonstrate similar adipogenic and chondrogenic differentiation potentials compared to ADSCs (Tateno et al., 2019).

### 4.2 ADSCs facilitate the repair of complex maxillofacial bone defects

Currently, autologous and allogeneic bone grafts remain the gold standard for repairing craniofacial bone defects (De Long et al., 2007). However, there are limitations such as insufficient bone quantity from donor sites, sequelae caused by bone defects in the donor area, unsuitability of donor bone morphology for craniofacial applications, and risks of infection and rejection associated with bone grafts (Smith et al., 2014). Therefore, it is urgent to find alternatives to bone for osteogenic differentiation. In this context, ADSCs have been identified as an excellent option for bone grafting (Barba et al., 2013). The use of expanded ADSCs can promote bone healing through direct differentiation into mature osteoblasts. ADSCs can secrete vascular endothelial growth factor (VEGF) (Colnot, 2005) and platelet-derived growth factor (PDGF) (Rehman et al., 2004), which play important roles in repairing fractures or bone defects (Ferrara, 2004; Hu et al., 2021). Lee injected ADSCs intramuscularly into bone defects and found increased Runx2 expression and significantly improved bone formation compared to the control group (Lee et al., 2010). The scaffold structure can direct the differentiation of ADSCs towards an osteogenic pathway rather than the more conventional adipogenic differentiation, ultimately leading to a more favorable outcome in bone repair (Fang et al., 2021; Tu et al., 2023). Sándor reported 13 cases of continuous craniofacial hard tissue defects repaired with the aid of ADCSs. In these cases, autologous adipose tissue was extracted from the anterior abdominal wall for culture expansion and then implanted into the hard tissue defects using scaffold materials. Among the 13 cases, 10 achieved complete bone reconstruction success, with ADSCs integrating into the surrounding bone. Only one patient experienced surgical failure due to poor habits (Sándor et al., 2014). In addition, ADSCs can promote the expression of TGF-β1 and directly synergize with TGF-β1 to enhance the migratory capacity of BMSCs, which has shown superior effects in the treatment of bisphosphonate-related osteonecrosis of the jaws. (Dong et al., 2021).

ADSCs possess paracrine capabilities and can secrete soluble factors and exosomes (Caplan and Correa, 2011). Therefore, researchers have considered whether exosomes can exert similar effects as ADSCs. Yang studied the expression profile of exosomal miRNAs and found that changes in exosomal miRNAs can promote osteogenic differentiation of ADSCs (Yang et al., 2019), which also demonstrates that exosomal miRNAs can regulate the osteogenic process of ADSCs. The emergence of tissue engineering has provided a broader scope for the application of exosomes derived from ADSCs (adipose-derived stem cells). By utilizing scaffolds that gradually degrade and enhance porosity to load exosomes, a continuous release of these vesicles can be achieved while creating an osteogenic microenvironment, thereby facilitating bone regeneration over extended periods (Gandolfi et al., 2020).

### 4.3 ADSCs utilize three-dimensional scaffold structures for skin wound repair

Surgeries for head and neck tumors, extensive facial burns, severe facial trauma, and other conditions can result in large areas of skin defects in the craniofacial region, which are difficult to repair spontaneously. Traditional autologous skin grafts are often insufficient and can leave new scars at the donor site. ADSCs (adipose-derived stem cells) autologous transplantation provides a new solution. ADSCs can regulate the regenerative microenvironment, secrete healing factors during wound repair (Reagan and Kaplan, 2011; Wendt et al., 2011; Anthony and Shiels, 2013), and have anti-inflammatory and immunosuppressive effects (Liu et al., 2015), thereby promoting cell proliferation and migration at the wound site (Maxson et al., 2012). ADSCs can also secrete growth factor-related proteins such as TGF-β1 and VEGF at the wound site, reducing scar formation (An et al., 2021). Tissue engineering technology has improved the survival environment of ADSCs at the wound site, further enhancing their ability to promote wound healing (Wu et al., 2021). Abbasi constructed a collagen/ADSCs hydrogel and implanted it into rat wounds, observing accelerated wound healing and significantly increased hair follicle and blood vessel formation (Abbasi et al., 2023). Zhang et al. modified and prepared a novel collagen sponge scaffold (NCSS), combined it with ADSCs, and implanted it into full-thickness skin wounds in nude mice. They found that the wound healing rate increased, and the vascular density around the wound significantly improved (Zhang A-J. et al., 2018).

In addition to promoting bone formation, ADSCs-derived exosomes also play a significant role in wound healing. Zheng found that exosomes expressing miR-378 can improve oxidative stress injury at the wound site and protect the wound (Zheng T. et al., 2021). Zhang further discovered that using ADSCs-derived exosomes, even without transplanting ADSCs and adopting a cell-free therapy approach, can still optimize collagen deposition through the PI3K/Akt signaling pathway, promote fibroblast proliferation and migration, and thus achieve the effect of promoting wound healing (Zhang W. et al., 2018).

### 4.4 Tissue engineering enables ADSCs to acquire the capability for periodontitis repair

Periodontitis often causes periodontal defects. Due to the presence of inflammation, the effects of bone grafting or induced osteogenic differentiation are limited. In an inflammatory environment, it is difficult for transplanted bone to grow or even survive, and osteogenic differentiation of stem cells closely related to bone is also significantly inhibited, which may even promote osteoclast genesis. The pluripotency and anti-inflammatory properties of ADSCs (adipose-derived stem cells) provide a new approach for the repair of periodontal defects (Liu et al., 2015). Sadeghi combined β-TCP with ADSCs and implanted them into periodontal defects in rats, observing not only new bone formation in the alveolar bone but also significant recovery of periodontal tissues (Sadeghi et al., 2014). The DFAT cells mentioned earlier also exhibit similar effects, capable of reconstructing periodontal tissue and promoting the accumulation of bone and cementum near the periodontal ligament. (Akita et al., 2016). Studies have also shown that PLGA scaffolds can help maintain the tissue regeneration space at periodontal defects due to their higher structural integrity compared to soft scaffolds such as hydrogels and sponge collagen. Transplanting DFAT cells or ADSCs onto PLGA scaffolds at periodontal defects can rapidly form connective tissue, aiding in the repair of periodontal defects (Doğan et al., 2003). Fujisaki successfully induced osteogenic differentiation using bone morphogenetic protein 2 (BMP-2), which enhanced the expression of osteogenic-related molecules in DFAT cells. Compared to ADSCs, transplantation of DFAT cells can better promote the formation of new mandibular bone *in vivo* through the ERK1/2 and Smad2 signaling pathways, providing a new strategy for jaw reconstruction (Akita et al., 2016).

## 5 Periodontal ligament stem cells and gingival mesenchymal stem cells

Periodontal ligament stem cells (PDLSCs) and gingival mesenchymal stem cells (GMSCs) are both unique mesenchymal stem cells specific to the periodontium in the oral cavity. Compared to other mesenchymal stem cells mentioned in the text, their sources are relatively uniform, both derived from periodontal tissues. Therefore, current research on them mainly focuses on oral applications, such as alveolar bone defect repair, bone resorption and formation during orthodontic tooth movement, and periodontitis. However, these two mesenchymal stem cells still exhibit promising osteogenic differentiation potential and possess unique characteristics compared to other mesenchymal stem cells. When combined with tissue engineering and appropriate scaffold materials, they may provide significant assistance in the repair and reconstruction of craniofacial defects in the future. Hence, we discuss them together in this context.

### 5.1 Tissue engineering enables PDLSCs and GMSCs to exert bone repair ability in craniomaxillofacial region

Periodontal ligament stem cells (PDLSCs) are mesenchymal stem cells derived from the periodontal ligament. Compared to other mesenchymal stem cells, such as BMSCs, PDLSCs exhibit higher growth potential and excellent multipotency. It has been demonstrated that PDLSCs possess the potential for chondrogenic, osteogenic, and adipogenic differentiation (Xu et al., 2009). PDLSCs are sensitive to tensile stress, and cyclic tensile stress can effectively promote their osteogenic differentiation (Shao et al., 2023). Meanwhile, the extracellular matrix can also enhance the osteogenic differentiation of PDLSCs at alveolar bone defect sites (Wen et al., 2019). However, this ability is primarily limited to the periodontal region, particularly affecting alveolar bone formation during orthodontic tooth movement. The osteogenic differentiation effect of PDLSCs is not significant in other craniofacial areas, making it challenging to apply them to the repair of craniofacial defects. Therefore, researchers have considered using three-dimensional scaffolds to provide a more suitable environment for osteogenic differentiation of PDLSCs, enabling them to exert their osteogenic potential in other regions. Cai et al. placed PDLSCs in a peptide-containing bioscaffold and observed increased bone formation while reducing chondrocyte generation (Cai et al., 2022). Zhao et al. co-cultured PDLSCs with umbilical vein endothelial cells (UVECs) in a calcium phosphate scaffold implanted into a cranial bone defect in a large animal model. They successfully promoted vascularization and bone formation in the defect site using the vascularized three-dimensional structure (Zhao et al., 2023). Hydrogel structures can also create a biologically active platform that satisfies the osteogenic effects of PDLSCs, facilitating their role in bone formation and repair of bone defects in the cranium (Isik et al., 2023). This brings hope for the application of PDLSCs in craniofacial defect repair. Future research directions may explore optimal scaffold materials and supplementary cytokines that enable PDLSCs to exert their multipotency in craniofacial regions beyond the periodontium.

Gingival mesenchymal stem cells (GMSCs) are mesenchymal stem cells derived from the gingiva. Compared to other mesenchymal stem cells, GMSCs are easily accessible, isolated from the gingival propria using minimally invasive techniques (Wang et al., 2011). Possibly due to the absence of a keratinized layer on the gingival surface, GMSCs maintain a relatively stable phenotype *in vitro* and can proliferate for extended periods. These characteristics make GMSCs a potential candidate for craniofacial defect repair among mesenchymal stem cells. Similar to other mesenchymal stem cells, GMSCs exhibit excellent tissue repair capabilities. GMSCs have significant osteogenic differentiation potential, expressing high levels of osteogenic markers (Tomar et al., 2010), suggesting their potential application in bone defect repair or bone regeneration. Wang et al. used type I collagen to transplant GMSCs into rat mandibular bone defects, demonstrating that GMSCs can directly participate in bone repair at the defect site. They also recruit BMSCs by secreting trophic factors, further promoting bone regeneration. This may be because the proportion of CD90-positive cells in GMSCs is much higher than in BMSCs (Sun et al., 2019). The recruited BMSCs by GMSCs have a higher proportion of CD90-positive cells than conventional BMSCs, thus exhibiting better osteogenic potential.

### 5.2 PDLSCs promote nerve repair

Severe craniofacial injuries are often associated with multiple nerve damages, resulting in limited function despite the reconstruction of bones and muscles, accompanied by symptoms such as facial numbness. Nerve cells have weak growth ability, requiring a long recovery time after injury, and the recovery effect is often unsatisfactory. The mesenchymal stem cells mentioned earlier have weak neurogenic differentiation potential, which cannot meet the demand for craniofacial nerve repair. PDLSCs partially originate from neural crest-derived cells, while nerve cells also originate from the neural crest. This suggests that PDLSCs are more likely to possess the ability to differentiate into nerve cells compared to other mesenchymal stem cells, which may have significant implications in craniofacial reconstruction. Wdiera et al. isolated somatic stem cells from human periodontium and successfully achieved neurogenic differentiation of PDLSCs in a culture medium containing epidermal growth factor (EGF) and fibroblast growth factor-2 (FGF-2) (Widera et al., 2007). After inducing neurogenic differentiation in this manner, the expression of b-tubulin III and nestin increased significantly, and sodium ion channels exhibited inward and outward currents, indicating the presence of neural connections. This suggests that PDLSC-differentiated cells can truly perform the functions of nerve cells. Furthermore, TK et al. used PDLSCs from adults to differentiate into retinal ganglion-like cells (Ng et al., 2015), demonstrating the feasibility of PDLSCs’ neurogenic differentiation potential in craniofacial reconstruction and repair. Additionally, PDLSCs can secrete neurotrophic factors through paracrine signaling, protecting nerve cells, promoting axon regeneration and synapse formation, and aiding in the recovery and growth of nerve ganglia (Tomokiyo et al., 2012). In conclusion, the combination of these two approaches may contribute to craniofacial repair and reconstruction, further restoring the sensory system and fine motor skills of the craniofacial region, and improving patients’ quality of life.

### 5.3 GMSCs have advantages in wound repair

Wounds in the gingiva heal quickly with a very low incidence of complications. This characteristic led researchers to investigate whether GMSCs from the gingiva could aid in the repair of tissue wounds beyond the gingiva. Surprisingly, GMSCs can induce M2 macrophage polarization while inhibiting the production of inflammatory factors such as IL-6 and TNF-α, significantly accelerating wound healing (Zhang et al., 2010). Additionally, the stable phenotype, strong proliferative ability *in vitro*, and robust self-renewal capacity of GMSCs make them highly suitable for repairing wounds of various sizes. Furthermore, when TGF-β structural microspheres carry GMSCs into the body, they can specifically express genes such as Scx, DCn, and Bgy, promoting tendon regeneration (Moshaverinia et al., 2014). This has exciting implications for preventing and reducing scar formation.

## 6 Induced pluripotent stem cells

Induced pluripotent stem cells (iPSCs) are a type of pluripotent stem cells derived from terminally differentiated somatic cells through reprogramming with specific transcription factors. Yamanaka first generated iPSCs by transducing four transcription factor genes, Oct3/4, Sox2, c-Myc, and Klf4, into mouse embryonic fibroblasts using retroviral vectors, successfully converting them into pluripotent stem cells (Takahashi and Yamanaka, 2006). The use of iPSCs in craniofacial reconstruction is significant due to their derivation from the donor’s own somatic cells, which greatly reduces the risk of immune rejection. Additionally, iPSCs do not originate from embryonic or oocyte cells, thus avoiding ethical issues and broadening their applicability. The pluripotency of iPSCs makes them highly versatile in craniofacial reconstruction, not only contributing to the regeneration of craniofacial bones but also playing a crucial role in the reconstruction of soft tissues such as muscles, skin, and blood vessels (Kato et al., 2022). The ability of iPSCs to differentiate into various cell types makes them a promising tool for craniofacial reconstruction, with the potential to improve surgical outcomes and patient quality of life (Table 3).

**TABLE 3:** The authors, materials, experimental subjects, experimental models, types of experiments, sources of mesenchymal stem cells, effects, and follow-up time of the literature on tissue engineering related to ADSCs.

Author	Scaffold	Research subjects and quantity	Diseases	Study design	MSCs source	Outcomes	Follow-up
Rufaihah et al. (2011)	polymer or polymer/calcium silicates composite scaffolds	mice	Oral cavity bone defect	Animal experiments	h-ADSCs	Improved bone regeneration	28 days
Ren et al. (2022)	β-TCP	Rat	Alveolar bone defect	Animal experiments	Autologous	Increase in alveolar bone volume and restoration of periodontal tissues	
Bilousova et al. (2011)	NCSS	nude mice 24	Full-thickness skin defect	Animal experiments	Autologous	Enhanced wound healing rate and increased vascular density	
Schlabe et al. (2008)	collagen/ADSCs hydrogel	Rat 12	Skin defect	Animal experiments	Autologous	Enhanced wound healing rate with increased hair follicle and vascular formation	14 days
Qin et al. (2004)	Chitosan/Nanohydroxyapatite-P24 Nanocomposite	Rat 36	Osteoporosis	Animal experiments	Autologous	Promoted bone regeneration and inhibited bone resorption	8 weeks
Park et al. (2022)	cell sheet	Rat 32	mandible defect	Animal experiments	Autologous	Promoted bone regeneration	8 weeks
Hibino et al. (2012)	MTG	Rat	diabetic periodontal wounds and craniofacial defects	Animal experiments	Autologous	Accelerated wound healing of oral mucosa and regeneration of diabetic bone defects	28 days
Panaroni et al. (2014)	bilayer biodegradable PLA	Rabbits 7	Temporomandibular joint disc with impairment	Animal experiments	Autologous	Reconstructed the temporomandibular joint disc	6 weeks
Sood et al. (2010)	integral ADSCs-NO hydrogel scaffolds	Mice	Severe burn	Animal experiments	Autologous	Promoted wound healing	14 months

β-TCP, Beta-tricalcium phosphate; NCSS, new collagen sponge scaffold; mTG, microbial transglutaminase cross-linked gelatin hydrogel; PLA, polylactic acid.

### 6.1 iPSCs bring new insights for the repair of comminuted fractures

When craniomaxillofacial bone defects exceed the critical size for repair capabilities, such as in cases of comminuted fractures, open fractures, or after surgical removal of bone tumors, autologous bone cannot complete the repair and reconstruction, and additional external bone is required (Umrath et al., 2020). Traditionally, additional osteoblasts have been obtained mostly by extracting bone marrow mesenchymal stem cells (BMSCs) from surgically removed bone fragments (Panaroni et al., 2014). However, the number of BMSCs extracted in this way is limited by the amount of bone removed, and there are issues with low survival rates and a gradual decline in proliferative and differentiation potential (Meijer et al., 2007; Dimitriou et al., 2011). iPSCs transplantation may be a feasible direction to solve the above problems (Zhang et al., 2016). The utilization of three-dimensional scaffolds to encapsulate iPSCs for *in vivo* delivery, or the induction of chondrogenic differentiation of iPSCs *in vitro* followed by their transplantation via scaffolds, can both achieve cartilage regeneration and the generation of new chondrocytes at the defect site (Liu et al., 2014; He et al., 2016). Compared to the control group, significantly more new bone was observed at the site of implantation of iPSCs-derived chondrocytes, with both osteogenic and chondrogenic trends (Iimori et al., 2021). However, currently, there is no evidence that iPSCs have a clear role in promoting bone maturation. Most experiments have shown that although the number of osteoblasts increases and the osteogenic trend improves after adding iPSCs, there is no significant increase in the final amount of mature bone (Repic et al., 2013), which may be related to the steady state of osteogenic and osteoclastic genes. The specific mechanism still needs to be further explored.

The treatment of craniomaxillofacial defects or developmental disorders caused by gene mutations is often difficult because the changes in genes lead to dysfunction of the original osteoblasts and chondrocytes, and even with surgical repair, the prognosis is not ideal. Ooki demonstrated that activation of the Runx2 promoter is closely related to osteoblast differentiation (Ooki et al., 2020). Haploinsufficiency of Runx2 can lead to craniosynostosis (CCD), often accompanied by significant osteoporosis (Liu et al., 2001), which poses challenges for subsequent craniomaxillofacial reconstruction. The current conventional treatment method is mesenchymal stem cell (MSC) transplantation therapy, but defects in the Runx2 gene limit the osteogenic differentiation capacity and number of transplanted MSCs, resulting in poor craniomaxillofacial reconstruction outcomes. iPSCs technology provides a new solution for these patients. It can either use Runx2 gene-normal iPSCs to differentiate into osteoblast lineage cells *in vitro* (Zhu et al., 2019), or establish reversible iPSCs using programmable nucleases and clustered regularly interspaced short palindromic repeats (CRISPR)/Cas-derived RNA-guided endonucleases *in vitro* to correct mutant Runx2 genes in CCD-iPSCs (Saito et al., 2018). Transplantation and osteogenic induction at mouse bone defect sites have shown that iPSCs improve the differentiation of originally abnormal osteoblasts and significantly enhance bone healing effects. Chubb introduced iPSCs into Runx2-deficient blastocyst embryos, successfully increasing the number of hematopoietic stem cells and restoring hematopoietic and osteogenic capabilities in the bone marrow.

### 6.2 iPSCs and their paracrine products promote vascular remodeling

The blood supply to the maxillofacial region is primarily provided by multiple branches of the external carotid artery, which interweave to form a dense vascular network, ensuring adequate blood supply to this area. Specifically, the lingual artery, external maxillary artery, internal maxillary artery, and superficial temporal artery are the main blood supply branches (Park et al., 2022). Among them, the external maxillary artery, as a key branch of the external carotid artery, can be palpated for a prominent arterial pulse at the junction of the anterior border of the masseter muscle and the inferior border of the mandible, providing an effective compression point for clinical management of bleeding in the mid-lower facial region. This rich blood supply endows the maxillofacial tissues with strong regenerative and anti-infectious capabilities, but also implies a potential for significant blood loss in cases of trauma. Additionally, the maxillofacial region is prone to vascular diseases, with benign vascular diseases being one of the most common pathologies in infant soft tissues, such as arteriovenous malformations (Qin et al., 2004; Zhang WX. et al., 2023).

Due to the lengthy and often insufficient process of endothelial cell regeneration, the unlimited proliferative potential and pluripotency of induced pluripotent stem cells (iPSCs) hold significant value in vascular reconstruction. Numerous animal experiments have demonstrated that iPSC-derived vascular endothelial cells can engraft in mouse arteriovenous chambers and exert vasculogenic effects (Stamm et al., 2010; Hibino et al., 2012; Dash et al., 2015).

The technology of tissue engineering has significantly enhanced the angiogenic potential of iPSCs. Scaffolds with specific structures or compositional elements can notably improve the angiogenic capacity of iPSCs both *in vitro* and *in vivo*. For instance, the utilization of a three-dimensional silicate bioceramic structure for delivering iPSCs facilitates osteogenesis while concurrently promoting vasculogenesis *in vivo* (Dong et al., 2019). However, the scaffold structure can also impede the tight integration of iPSCs with blood vessels, affecting their survival rate and angiogenic capacity. In such scenarios, biodegradable scaffolds emerge as a viable option. Following the transplantation of a vascular graft combining iPSCs with the scaffold into the vascular site, the degradation of the scaffold allows unhindered angiogenic activity of the iPSCs (Luo et al., 2020), presenting a novel approach for vasculogenesis.

Rufaihah transplanted endothelial cells (ECs) extracted from human iPSCs into a mouse model of peripheral arterial disease via intramuscular injection, observing a significant increase in capillary density and blood perfusion in the previously ischemic areas at the injection site. Rufaihah suggests that iPSC-derived ECs not only integrate with blood vessels but also exert paracrine effects to promote angiogenesis (Rufaihah et al., 2011). However, due to concerns about the tumorigenic risks associated with iPSCs (Kuroda et al., 2013), there have been no clinical trials applying iPSCs to craniofacial vascular reconstruction. Perhaps in the future, when the tumorigenic risks of iPSCs are reduced, there will be greater opportunities for their application. The use of iPSC-derived exosomes for therapy may be a promising direction. Zhang et al. discovered that iPSC-derived exosomes possess angiogenic properties that promote wound healing, along with the advantage of low tumorigenicity (Zhang J. et al., 2023). Additionally, the utilization of iPSCs in manufacturing functional and contractile vascular smooth muscle cells has already demonstrated significant value. They not only contain disease-causing mutations but also, in many cases, possess the permissive genetic background required for full disease expression (Ge et al., 2012; Sinha et al., 2014), making iPSCs highly suitable for vascular disease model reconstruction.

### 6.3 iPSCs promote wound healing and skin regeneration

The appearance of the maxillofacial region and the function of its muscles are crucial, making skin wounds and scar repair in craniomaxillofacial reconstruction even more important than in other areas. The current common treatment approach involves the use of tissue-engineered skin for rapid epithelialization, restoration of skin barrier function, and promotion of wound healing (Yanaga et al., 2001; Sood et al., 2010; Do and Eming, 2016). However, this method is limited by the survival rate of transplanted cells and immune rejection. Thanks to the pluripotency and autologous origin, induced pluripotent stem cells (iPSCs) offer a new strategy for craniomaxillofacial skin wound healing (Schlabe et al., 2008). Both Bilousova and Kidwai have successfully used iPSCs to induce keratinocytes that can be used in clinical settings (Bilousova et al., 2011; Do and Eming, 2016). Based on this, Yan directly injected iPSCs-derived keratinocytes into skin wounds and found that it could accelerate wound healing and increase the trend of re-epithelialization. Yan believed that this could be due to the anti-fibrotic ability exerted by secreting TNF-α. As the injected iPSCs are located in the subcutaneous tissue and need to migrate to the wound edge for wound healing, scholars have further explored how iPSCs-derived keratinocytes migrate from the injected subcutaneous tissue to the wound edge for epithelialization. They found that the adhesion of extracellular matrix (ECM) to iPSCs reduces their migratory capacity (Fuchs et al., 2004; Polisetti et al., 2016). To address this issue, both reducing adhesion and enhancing migratory capacity are viable solutions. If the Itgb1 gene in iPSCs is knocked out to reduce adhesion to the ECM, wound healing can be accelerated, and the survival rate of transplanted skin can be improved (Ren et al., 2022). On the other hand, some proteins such as heat shock protein-90α (Hsp-90α) can activate the Akt signaling pathway, promoting the migration of iPSCs-derived keratinocytes towards the edges of burn wounds (Wu et al., 2019). Exosomes produced by iPSCs-derived keratinocytes can also enhance the migration of endothelial cells and keratinocytes by secreting miR-762, promoting angiogenesis and re-epithelialization (Bo et al., 2022), and accelerating the healing of burn wounds. In summary, iPSCs possess pluripotency, low immunogenicity, high survival rate, and can be easily applied to skin wounds in a relatively simple way, providing a new strategy for maxillofacial skin reconstruction and repair.

### 6.4 Pathogenicity of tumors from iPSCs

While iPSCs hold promising potential for craniofacial repair and reconstruction, the risk of tumorigenicity associated with viral vector integration requires cautious consideration (Cavazzana-Calvo et al., 2010). Yamanaka, following the discovery of iPSCs, further found that reactivation of the c-Myc transgene during the induction process significantly increases the risk of tumor development (Okita et al., 2007; Nakagawa et al., 2008). Silencing of the tumor suppressor gene p53 leads to enhanced reprogramming efficiency, activation of proto-oncogenes, and reprogramming of genetically defective and uncontrolled cells that would normally undergo apoptosis, thereby greatly increasing tumorigenic potential (Hong et al., 2009). Cavazzana-Calvo identified that the involvement of iPSCs can cause HMGA2 gene dysregulation due to integrating vectors (Cavazzana-Calvo et al., 2010). Hacein-Bey-Abina reported two cases where retroviral enhancement drove the LMO2 gene following iPSC treatment, resulting in uncontrolled clonal proliferation of mature T-cells (Hacein-Bey-Abina et al., 2003). Other factors such as Nanog protein, a diagnostic marker and therapeutic target for lung cancer, and Oct4, which is primarily expressed in germline stem cells and various tumor cells (Deng et al., 2020), are also implicated. The C-terminus of Klf4 contains three tandem zinc finger sequences that play a critical role in activating the target gene Nanog, and Klf4 also serves as a regulator of Nanog. These findings suggest an intersection between iPSCs and tumor-related gene expression regulation. iPSCs may also lead to the deletion or rearrangement of tumor suppressor genes or activate proto-oncogenes, resulting in a significantly increased risk of cancer (Hacein-Bey-Abina et al., 2008).

On the other hand, prior to transplanting iPSC-derived cells into the body for therapy, multiple passages are required to expand the iPSCs *in vitro*. During this process, iPSCs with DNA repair defects may emerge (Hyka-Nouspikel et al., 2012). Although apoptosis regulation can respond to DNA damage, Desmarais found that a small subset of genetically mutated cells in iPSCs continues to proliferate (Desmarais et al., 2012), leading to an increasing number of variant cells in subsequent iPSC-derived populations, including chromosomal translocations, large-scale duplications and deletions, and point mutations (Mayshar et al., 2010). These cells are at high risk for subsequent malignant transformation.

To address the tumorigenicity barriers to clinical applications of iPSCs, researchers are continuously seeking methods to reduce tumorigenicity. Currently, some researchers have adopted strategies such as reducing reprogramming factors (Stadtfeld et al., 2008; Yu et al., 2009), screening for safer expression vectors (Li et al., 2011), utilizing reprogramming chemical small molecules to reduce iPSC tumorigenicity (Woltjen et al., 2009), and utilizing exosomes from iPSC-derived cells to exert similar functions, as mentioned earlier. The underlying causes and mechanisms of iPSC tumorigenicity are not fully understood; however, with continuous exploration by researchers and advances in retroviral technology, we can envision a future where iPSCs are applied in clinical craniofacial reconstruction.

## 7 Discussion

**FIGURE 2:**
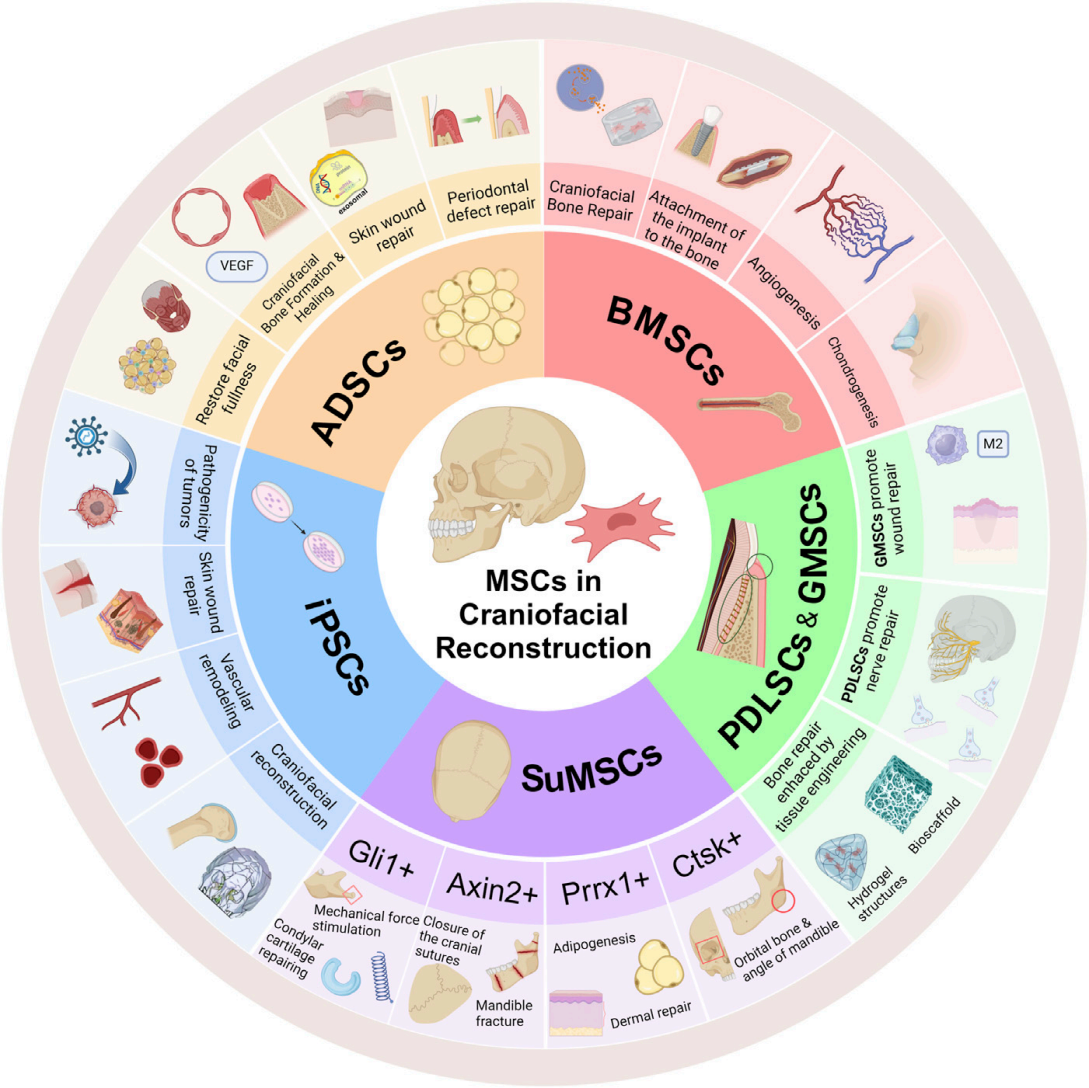
Different MSCs play distinct roles in craniofacial reconstruction. BMSCs have a significant promoting effect on the formation of various types of bone and cartilage, and can promote angiogenesis to ensure the survival of transplanted cells in tissue engineering. SuMSCs expressing different genes play different roles in craniofacial reconstruction, acting in areas such as bone formation, cartilage formation, adipogenesis, normal closure of cranial sutures, and skin formation. iPSCs, due to their pluripotency, play an important role in reconstructing comminuted fractures, forming cartilage, angiogenesis, and wound healing, but there is a risk of tumorigenicity. ADSCs have a unique role in the reconstruction of craniofacial fat, periodontal defects caused by periodontitis, and wound repair (Created with BioRender.com).

The reconstruction of craniofacial structures faces numerous challenges, including high complexity, specificity, severe injury, irregular and complex wounds, and a high risk of hemorrhage. Traditional bone grafts have not demonstrated satisfactory results in craniofacial reconstruction. The utilization of mesenchymal stem cells has provided new insights for craniofacial reconstruction. SuMSCs expressing specific genes play a unique role in the skull, particularly at suture sites. Regulation of specific genes in these SuMSCs expressing skull-specific genes may enable bidirectional modulation of skull development, potentially addressing issues such as craniosynostosis and premature skull suture closure. iPSCs offer a promising opportunity for more effective treatment of severe craniofacial injuries. Their high differentiation capacity and multilineage potential are crucial in craniofacial comminuted fractures or large wounds, promoting both new bone formation and fracture healing, as well as vascular anastomosis and wound recovery. Importantly, the autologous nature of iPSCs minimizes the risk of immune rejection, which is a significant benefit for patients with severe craniofacial defects. However, the retroviral transfection method used to generate iPSCs poses a risk of tumorigenicity, increasing the chance of cancer development in patients after surgery. This is a critical challenge that needs to be addressed before iPSCs can be applied in clinical trials. Currently, researchers are exploring various strategies to reduce the tumorigenic potential of iPSCs for clinical use, such as reducing reprogramming factors, using safer expression vectors, chemical reprogramming with small molecules, and utilizing iPSCs-derived exosomes as an alternative to iPSCs. In the nearly future, iPSCs may be officially utilized in clinical settings. ADSCs and DFAT cells demonstrate remarkable multipotency in the craniofacial region. Contrary to the traditional belief that they primarily undergo adipogenic differentiation, ADSCs and DFAT cells exhibit surprising capabilities in osteogenesis, chondrogenesis, and vasculogenesis. Therefore, ADSCs may be the most suitable mesenchymal stem cells for craniofacial reconstruction. However, there are still many unanswered questions, such as the underlying causes and mechanisms of iPSCs tumorigenicity and the mechanisms behind ADSCs promoting osteoclastogenesis or osteogenesis in different craniofacial regions. These could be potential areas for further in-depth research (Figure 2).
